# Candidate loci for phenology and fruitfulness contributing to the phenotypic variability observed in grapevine

**DOI:** 10.1007/s00122-013-2170-1

**Published:** 2013-08-06

**Authors:** Lukasz Grzeskowiak, Laura Costantini, Silvia Lorenzi, M. Stella Grando

**Affiliations:** Centre for Research and Innovation, Fondazione Edmund Mach (FEM), San Michele all’Adige, Italy

## Abstract

*****Key message***:**

**In this study, we identified several genes, which potentially contribute to phenological variation in the grapevine. This may help to maintain consistent yield and suitability of particular varieties in future climatic conditions.**

**Abstract:**

The timing of major developmental events in fruit crops differs with cultivar, weather conditions and ecological site. This plasticity results also in diverse levels of fruitfulness. Identifying the genetic factors responsible for phenology and fertility variation may help to improve these traits to better match future climates. Two *Vitis vinifera* populations, an F1 progeny of Syrah × Pinot Noir and a phenological core collection composed of 163 cultivars, were evaluated for phenology and fertility subtraits during three to six growing seasons in the same geographical location. The phenotypic variability in the core collection mostly overlapped with that observed in the F1 progeny and several accessions had exceeding values of phenological response. The progeny population was used together with SSR and SNP markers to map quantitative trait loci (QTLs). This allowed us to detect nine QTLs related to budburst, flowering beginning, the onset of ripening (*véraison*) and total fertility, explaining from 8 to 44 % of phenotypic variation. A genomic region on chromosome 15 was associated with budburst and *véraison* and two QTLs for fruitfulness were located on chromosomes 3 and 18. Several genes potentially affecting fertility and the timing of fruit development were proposed, based on their position and putative function. Allelic variation at these candidate loci may be explored by sampling accessions from the core collection.

**Electronic supplementary material:**

The online version of this article (doi:10.1007/s00122-013-2170-1) contains supplementary material, which is available to authorized users.

## Introduction

Phenological differences among genotypes may affect the majority of biological phenomena, such as plant germination, flowering and pollination, fruit ripening, colour changing and leaf fall, as well as animal migration and breeding. From individual physiology to global metabolic changes, with regard to interspecific relationships, all these processes have periodic cycles and are influenced by the timing of environmental events (Schnelle 1955; Lieth 1974; Sparks and Menzel 2002; Wilczek et al. 2010). Recently, there has been an increasing interest in how alterations of phenology may affect plant adaptation to environment, as a number of studies have documented phenological responses to global climate change, which has also effects on human activities such as agriculture, forestry and viticulture (e.g. Chuine et al. 2004; Jones et al. 2005; Webb et al. 2007; van Leeuwen et al. 2008; Caffarra and Eccel 2011; Chew et al. 2012).

With regard to viticulture, there is a varying degree of phenotypic plasticity in grapevine phenology (Sadras et al. 2009; Dal Santo et al. 2013a). The key developmental stages, budburst, flowering and timing of harvest, are driven mainly by temperature and differ greatly with variety, climate and geographical location. For instance, the cultivar Pinot Noir can ripen together with Syrah in cold regions, but earlier than Syrah in warm regions (Dry 1983; Coombe 1988; van Leeuwen et al. 2008; Jackson 2008). This plasticity results also in different levels of fertility (fruitfulness) and yield (Sadras et al. 2009; Nicotra et al. 2010; Anderson et al. 2012). Several studies have shown that the growth rate and composition of the grape can be affected by climate change, representing a risk to present and future fruit production (e.g. Coombe 1988; Schultz 2000; Duchêne and Schneider 2005; Jones et al. 2005; Brunet et al. 2007; Jackson 2008; Webb et al. 2008; Duchêne et al. 2010; Keller 2010). Therefore, to maintain suitability of particular varieties and consistency in yield and wine styles, grape growers need to consider altering the balance of cultivars from specific regions or developing new cultivars with improved traits to better match future climate conditions (Schultz 2000; Webb et al. 2010; Hannah et al. 2013).

Exploiting the phenotypic and genetic differences between grapevines may allow for successful grape cultivation over a range of climate types and provides possibilities for traditional breeding, as well as identification of target genes for marker-assisted selection (Martinez-Zapater et al. 2010). One way to identify the location of key genes with reference to specific markers and the sequenced genome is by discovering quantitative trait loci (QTLs), which indicate regions of a genome that contribute to trait variation. For instance, QTLs related to fertility, growth and phenology have been mapped from F1 segregating progenies obtained by crossing two grapevine cultivars, and this has been used to start identification of candidate genes (Costantini et al. 2008; Doligez et al. 2010; Duchêne et al. 2010). Although only a handful of these QTLs have been characterized, and their structure and interactions are complex (Doligez et al. 2010; Martinez-Zapater et al. 2010), the potential adaptive benefits of exploiting the variation in phenological response were recently demonstrated using the progeny of cultivars Riesling and Gewürztraminer (Duchêne et al. 2010, 2012).

In this study, we evaluated the phenological and fruitfulness variability within two populations of wine grapes planted in the same location: a germplasm core collection composed of different cultivated varieties and a segregating population derived from a cross between cultivars Syrah and Pinot Noir. Next, we performed QTL mapping in the biparental population and detected several regions in the grapevine genome correlated with the phenotypic variation in budburst, flowering, the onset of ripening (*véraison*) and total fertility. Finally, we proposed and discussed several candidate genes based on a Gene Ontology (GO) term enrichment and functional annotation analysis.

## Materials and methods

### Plant material and climatic description of the study site

The grapevine populations analysed in this study belong to the FEM grape germplasm collection located in San Michele all’Adige (Trentino, Italy). The first population is a *Vitis vinifera* “phenological core collection” (core P) composed of 163 various cultivar accessions (listed in supplementary Table S1), replicated five times and selected as the most representative samples of genetic and agro-morphological diversity (Emanuelli et al. 2013). The second population is a progeny derived from a cross between grapevine varieties Syrah and Pinot Noir. It comprises 170 F1 individuals which were used for linkage map construction (Costantini et al. in prep). All plants of these two populations were grafted on the rootstock Kober 5BB at the FEM experimental field “Giaroni”, and then uniformly pruned and trained according to the Guyot system.

San Michele all’Adige has a humid continental climate classified as Dfb (snow, fully humid, warm summer) under the Köppen-Geiger climate classification system (Kottek et al. 2006). Weather records were obtained from a meteorological station located close to the FEM experimental field (elevation 205 m above sea level, 46.189° N, 11.134° E). The station recorded daily observations of maximum, minimum, and average temperatures and precipitation in 2005–2011 (supplementary Table S2). Climate characteristics for this period showed that precipitation averaged 90 mm during the growing season with a minimum of 68 mm in 2006 and a maximum of 131 mm in 2008. The average growing season temperature in the region was 18.1 °C (17–18 °C within all years). The average maximum temperature during the growing season was 23.2 °C (22.2 °C in 2008 and 24.8 °C in 2006). The average minimum temperature during the growing season was 11.8 °C with a low of 10.3 °C in 2010 to a high of 12.9 °C in 2006.

### Phenotypic assessment

Six developmental stages were defined based on the modified E-L system for grapevine phenological classification (Coombe 1995). These stages comprised: budburst (BB, stage E–L 4), when 50 % of the shoots had the leaf tips visible; beginning of flowering (FB, stage E–L 20) corresponding to 10 % of flower caps off; end of flowering (FE, stage E–L 26), when flower cap fall was complete; beginning of *véraison* (VB, stage E–L 34), when berries started to soften; end of *véraison* (VE stage E–L 37) in which all berries were soft; and ripening (R, stage E–L 38), when juice extracted from the berries had 18 degrees Brix (a measurement of the sugar content in a solution). Phenotypic assessment was recorded from 3 to 6 years during 2005–2011, depending on trait and population analysed.

Plant observations and the daily climate data were summarized for the growing season from April to October, since growing season averages are adequate to explain the phenology of grapevine (Jones et al. 2005). The dates of each phenological stage were converted into day of the year (DOY), i.e. the number of days after January 1 on which the plant attained each developmental stage. For the progeny population, we calculated also the temperature accumulated over time, referred to as heatsum, which determines the rate of spring development of plants. Heatsum is the accumulation of growing degree days (GDD) up to the date of a phenological event. One GDD is equal to one degree above the base temperature during 24 h. A commonly used heat accumulation index is the Winkler index, where GDD is the sum of the differences between the mean daily air temperature and 10 °C threshold temperature over the active period from April to October (Amerine and Winkler 1944; Hunter and Lechowicz 1992; Ghelardini et al. 2006).

Berry clusters were counted during stage E–L 29 to calculate bud fruitfulness, i.e. the first measure of yield potential, expressed as the total fertility index and estimated by dividing the total number of clusters by the total number of growing shoots per plant. The fertility, phenology and climate data were analysed using the program SPSS 17.0 (SPSS Statistics for Windows, Version 17.0, SPSS Inc., Chicago, IL, USA).

### QTL analysis

For QTL identification, we used a previously constructed genetic linkage map based on the genotyping of 652 SSR and SNP markers in 170 F1 individuals from the progeny of Syrah and Pinot Noir (Costantini et al. in prep., updated from Troggio et al. 2007). In the case of the phenological traits, two types of phenotypic datasets were used for each year: one based on DOY and the other based on GDD. Exemplary correlations between DOY and GDD for 3 years (2008–2010) are shown in supplementary Fig. S1. The QTL analysis was performed in MapQTL 6.0 (van Ooijen 2009) with the simple interval mapping and multiple QTL mapping (MQM) functions (with step size 1 cM). Using these methods, background markers were selected to take over the role of the putative QTL as cofactors and reduce the residual variance. The LOD profiles from interval mapping were inspected and the marker closest to each LOD peak was selected as the cofactor to perform the MQM mapping. Several cycles were performed to obtain the potentially maximum number of cofactors for the MQM analysis. These cofactors were then subjected to backward elimination procedure, which leaves out one cofactor at a time to create a subset of markers. The retained set of cofactors was used for further rounds of MQM. In the final LOD profile, QTLs were declared significant, if the maximum LOD exceeded the linkage group and/or genome-wide LOD threshold (calculated using 1,000 permutations) and mean error rate was lower than 0.05. Each QTL was characterized by its LOD score and percentage of phenotypic variation explained in the mapping population. Further, a non-parametric Kruskal–Wallis test was performed to provide support to the marker–trait associations separately for each season. This test is regarded as the non-parametric equivalent to the one-way analysis of variance and indicates that the results of the QTL mapping are not influenced by segregation distortion or non-normal distribution of particular traits (Lehmann 1975; van Ooijen 2009). Confidence intervals were estimated in cM and corresponded to an LOD score drop of one on either side of the likelihood peak. The physical positions of these intervals are provided relative to the genome sequence of Pinot Noir clone ENTAV115 (Velasco et al. 2007).

### Candidate gene selection

We selected candidate genes from among functionally annotated genes located within three QTL intervals on chromosomes 3, 15 and 18. The reference genome PN40024 (12× assembly) was used to extract version 1 of the gene predictions (12×v1; Jaillon et al. 2007; Forcato 2010; Grimplet et al. 2012). The physical positions of the QTL intervals based on this reference genome were as follows (in bp): for chromosome 3: 6,686,683–8,843,323, for chromosome 15: 13,060,296–16,414,837 and for chromosome 18: 10,665,387–13,879,246. To choose the candidate genes the intervals were tested for GO annotation enrichment using agriGO (Du et al. 2010). The statistical significance of functional enrichment within the intervals was evaluated using the hypergeometric distribution. A GO term was significantly enriched in the QTL interval, if the *p* value was less than 0.05 in comparison with the dataset of all gene transcripts annotated in the reference genome (available in agriGO). The genes were grouped into eight functional categories: cellular process, development, metabolism, regulation, response to stimulus, signalling, transport and diverse functions (supplementary Tables S3, S4 and S5).

## Results

### Distribution of the phenological and fertility traits in the core collection and the QTL mapping population

**Fig. 1:**
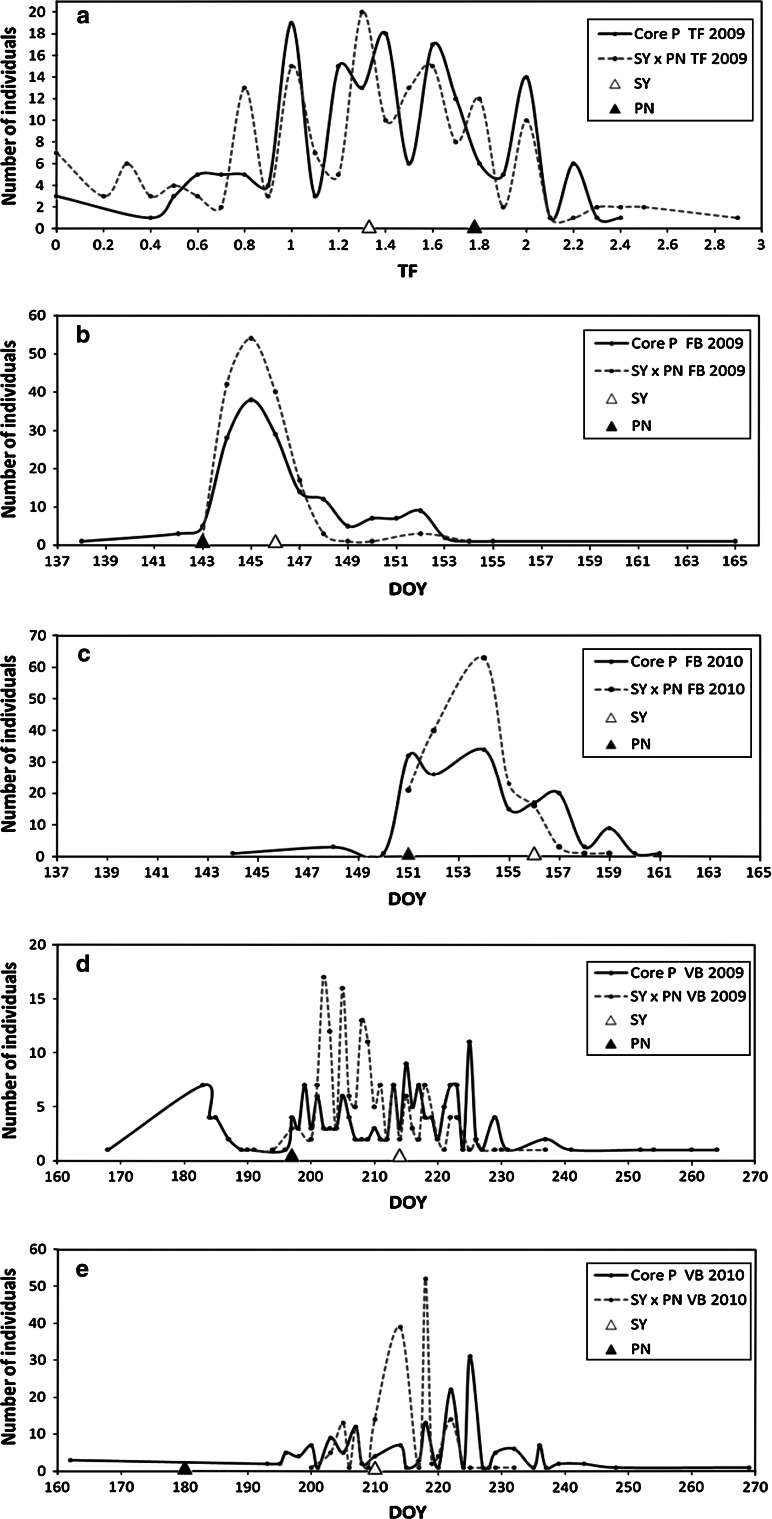
Comparison of phenotypic variability in two grapevine populations: the “phenological core collection” (Core P) and the progeny of a cross between Syrah and Pinot Noir (SY × PN). Exemplary frequency plots for: **a** total fertility index (TF) in 2009 and **b**–**e** the timing of flowering beginning (FB) and *véraison* beginning (VB) in 2009 and 2010, respectively; *DOY* day of year in Julian days

We observed non-normal distribution of the data for all traits and seasons, except for total fertility in 2009 (in both populations; Fig. 1a). The six phenological events defined in this study varied between cultivars of the “phenological collection” (core P), as well as in the progeny of the Syrah × Pinot Noir mapping population (SY × PN). The window of time in which each event typically occurred in the phenological collection among the analysed years was greater for *véraison* beginning and *véraison* end (both 75 days), as well as ripening (64 days), than for budburst (22 days), flowering beginning (19 days) and flowering end (16 days; Table 1). These values in the mapping population were as follows: 17 days for budburst, 12 days for flowering beginning, 14 days for flowering end, 39 days for *véraison* beginning, 47 days for *véraison* end and 31 days for ripening. Syrah was the earlier parent for budburst, while Pinot Noir was the earlier parent for flowering, *véraison* and ripening. Most of the progeny showed on average the timing of phenological events situated between both parents, but transgressive segregation was observed as well (Fig. 1b–e).

**Table 1:** Descriptive statistics and comparison of the phenotypic data from the grapevine “phenological core collection” (Core P) and the F1 progeny of Syrah and Pinot Noir (SY × PN) gathered during the same years (the timing of main phenological stages in DOY-days of year)

Trait	Years	Core P									SY × PN								
		*N*	Min		Max		Range	Mean		SD	*N*	Min		Max		Range	Mean		SD
BB	2008	162	96	Apr 07	123	May 02	27	109	Apr 18	5	168	100	Apr 09	117	Apr 26	17	110	Apr 19	4
	2009	162	93	Apr 03	114	Apr 24	21	102	Apr 11	4	168	96	Apr 06	114	Apr 24	18	100	Apr 10	3
	2010	161	99	Apr 09	116	Apr 26	17	106	Apr 16	4	169	99	Apr 09	116	Apr 26	17	106	Apr 16	4
							22	106	Apr 16							17	106	Apr 16	
FB	2006	163	147	May 27	167	Jun 16	20	157	Jun 06	4	148	148	May 28	165	Jun 14	17	156	Jun 05	2
	2007	162	134	May 14	155	Jun 04	21	141	May 21	3	167	131	May 11	142	May 22	11	139	May 19	4
	2008	162	145	May 24	165	Jun 13	20	156	Jun 04	4	166	151	May 30	162	Jun 10	11	155	Jun 03	2
	2009	162	138	May 18	155	Jun 04	17	146	May 26	3	164	143	May 23	154	Jun 03	11	145	May 25	2
	2010	161	144	May 24	161	Jun 10	17	154	Jun 03	3	168	151	May 31	159	Jun 08	8	154	Jun 03	2
							19	151	May 31							12	150	May 30	
FE	2006	163	163	Jun 12	174	Jun 23	11	168	Jun 17	2	148	155	Jun 04	174	Jun 23	19	167	Jun 16	2
	2007	162	145	May 25	162	Jun 11	17	152	Jun 01	5	167	134	May 14	156	Jun 05	22	150	May 30	3
	2008	162	153	Jun 01	168	Jun 16	15	162	Jun 10	2	166	158	Jun 06	165	Jun 13	7	160	Jun 08	1
	2009	162	141	May 21	160	Jun 09	19	153	Jun 02	3	164	147	May 27	160	Jun 09	13	150	May 30	2
	2010	161	151	May 31	167	Jun 16	16	159	Jun 08	3	168	154	Jun 03	163	Jun 12	9	157	Jun 06	2
							16	158	Jun 07							14	156	Jun 05	
VB	2006	163	190	Jul 09	253	Sep 10	63	220	Aug 08	14	148	204	Jul 23	239	Aug 27	35	223	Aug 11	7
	2007	162	183	Jul 02	262	Sep 19	79	207	Jul 26	16	166	194	Jul 13	221	Aug 09	27	208	Jul 27	6
	2008	161	186	Jul 07	263	Sep 19	77	219	Aug 06	14	166	188	Jul 06	241	Aug 28	53	220	Aug 07	7
	2009	162	183	Jul 02	264	Sep 21	81	211	Jul 30	15	160	190	Jul 09	237	Aug 25	47	209	Jul 28	8
	2010	160	193	Jul 12	269	Sep 26	76	218	Aug 06	13	166	200	Jul 19	232	Aug 20	32	214	Aug 02	6
							75	216	Aug 04							39	215	Aug 03	
VE	2006	163	204	Jul 23	276	Oct 03	72	243	Aug 31	16	148	228	Aug 16	247	Sep 04	19	241	Aug 29	5
	2007	162	199	Jul 18	261	Sep 18	62	233	Aug 21	17	167	201	Jul 20	236	Aug 24	35	222	Aug 10	8
	2008	161	203	Jul 21	281	Oct 07	78	241	Aug 28	19	164	212	Jul 30	274	Sep 30	62	238	Aug 25	12
	2009	161	197	Jul 16	276	Oct 03	79	233	Aug 21	23	159	198	Jul 17	267	Sep 24	69	227	Aug 15	15
	2010	160	198	Jul 17	284	Oct 11	86	232	Aug 20	18	165	207	Jul 26	258	Sep 15	51	226	Aug 14	9
							75	235	Aug 23							47	231	Aug 19	
R	2006	163	215	Aug 03	276	Oct 03	61	253	Sep 10	14	88	249	Sep 06	262	Sep 19	13	257	Sep 14	5
	2007	162	212	Jul 31	275	Oct 02	63	246	Sep 03	19	143	248	Sep 05	267	Sep 24	19	250	Sep 07	5
	2008	162	225	Aug 12	294	Oct 20	69	263	Sep 19	18	166	233	Aug 20	263	Sep 19	30	247	Sep 03	8
	2010	160	237	Aug 25	299	Oct 26	62	258	Sep 15	18	88	237	Aug 25	299	Oct 26	62	247	Sep 04	14
							64	256	Sep 13							31	250	Sep 07	
TF	2007	163	0.00		2.89		2.89	1.64		0.42	170	0.00		3.00		3.00	1.52		0.60
	2008	163	0.00		2.60		2.60	1.39		0.48	170	0.00		3.00		3.00	1.67		0.54
	2009	163	0.00		2.40		2.40	1.37		0.48	170	0.00		2.86		2.86	1.26		0.58
							2.63	1.47								2.95	1.48		

*BB* budburst, *FB* flowering beginning, *FE* flowering end, *VB* *véraison* beginning, *VE* *véraison* end, *R* ripening, *TF* total fertility index)

Further evaluation of these two populations in the analysed period of 3 to 6 years indicated that in core P budburst occurred on average on April 16, with a range between the earliest and the latest variety of 23 days (Magaratch and Garganega, respectively; supplementary Table S1). The highest year-to-year budburst variability was observed for varieties Datal, Goyura and Terret Noir (SD ± 8), and the lowest for Fertilia, Humagne, Morrastel, Neretta cuneese and Ortega (SD ± 1). In the SY × PN mapping population, the average budburst occurred also on April 16.

Flowering started in the phenological core collection on May 31 on average, with a range of 15 days (the earliest was Léon Millot and the latest Bombino bianco). The among-years variability in flowering beginning was the highest for Cegled szepe (SD ± 11) and the lowest for Beogradska Rana, Bombino bianco, Dunkelfelder, Kanzler Feld O, Maiolica, Petit meslier and Zweigelt blau (SD ± 4). In the SY × PN progeny, on average, flowering began on May 30.

Flowering ended in the core P on average on June 7, with a range of 13 days (the earliest was Léon Millot and the latest Airen, Alarije, Albana, Bombino bianco, Coda di volpe bianca, Parellada and Trebbiano Toscano). The highest year-to-year flowering end variability was observed for varieties Arnsburger, Ehrenfelser, Muscat delecta, Ortega, Sicilien and Turan (SD ± 9), and the lowest for Bombino bianco and Parellada (SD ± 4). In the SY × PN mapping population, flowering ended on average on June 5.

The average date of *véraison* beginning occurred in the observed 163 varieties on August 4, ranging for 68 days (the earliest accession was Turan and the latest Ohanés). The highest among-years variation of *véraison* beginning was observed for Malvasia di candia aromatica (SD ± 19) and the lowest for Coda di volpe bianca, Fertilia, Jacquère, Maiolica and Monja (SD ± 3). In the SY × PN mapping population, *véraison* started on average on August 3.

*Véraison* ended in the core collection on August 23 on average, with a range of 65 days (the earliest was Madelaine angevine × Calabrese and the latest Raboso Piave). The year-to-year variability in *véraison* end was highest for Ohanés (SD ± 28) and lowest for Léon Millot (SD ± 3). In the SY × PN progeny, *véraison* ended on August 19 on average.

Ripening (harvest) dates for the observed varieties of the phenological collection averaged on September 13, with a range of 54 days between the earliest variety Nektar and the latest variety Ohanés. The highest variation among years in this stage was observed for Cegled szepe and Léon Millot (SD ± 31), and the lowest for Petit Meslier (SD ± 4). Dates of harvest for the SY × PN progeny averaged on September 7.

Intervals between the main phenological events are also an important measure of developmental timing. The 163 grapevine varieties in core P revealed an average interval from budburst to flowering end of 54 days. The shortest interval between these two events was 44 days (in accessions Blauer Gelbhoelzer and Garganega) and the longest 60 days (in varieties Early Muscat and Perlon). The interval between budburst and *véraison* beginning was 110 days on average, with a range of 62 days from the shortest average interval of 88 days in varieties Beogradska Rana and Turan to 150 days in the variety Ohanés. The period from flowering beginning to *véraison* beginning averaged 64 days, with a range of 64 days for the analysed years (the variety Turan had the shortest average interval of 40 days, while Ohanés had the longest average interval of 104 days). The time from flowering beginning to ripening for the 163 cultivars averaged 102 days. This interval varied from the shortest for Beogradska Rana, Contessa and Nektar (77 days) to the longest for Aspiran noir and Ohanés (126 days). The total ripening stage from *véraison* beginning to harvest had an average of 38 days, with a range of 53 days. Coda di volpe bianca and Pinot meunier had the shortest average interval of 20 days, while Boglarka had an average 73 day interval. The length of the interval from budburst to ripening for the region studied covered the period from early April to late October and averaged 147 days across cultivars in the collection. This interval characterized the time needed for each plant to ripen and varied by 51 days on average (from 123 days for varieties Nektar, Pinot meunier and Sicilien to 174 days for Dattier noir).

The total fertility coefficient was estimated as the number of flower clusters per number of shoots. The average fertility index value in the phenological core collection was 1.47. The highest average fertility index value 2.2 was obtained for varieties Charmont, Schiras Samling and Segalin, and the lowest average fertility index value 0.39 was obtained for the variety Braghina. The highest year-to-year variability of this parameter was observed for cultivars Malvar and Montonico bianco (SD ± 1.0), and the lowest for Coarna neagra and Contessa (SD ± 0.0). In the mapping population, the average fertility index in these same growing seasons (2007–2009) was 1.48 and ranged from 0.33 to 2.53.

Spearman rank-order correlations between the analysed traits within each year were significant at *p* < 0.01 in the core P collection and at *p* < 0.05 in the SY × PN population. In general, correlations in the core collection were around twice higher than in the mapping population. The strongest correlations in both populations were between flowering beginning and flowering end, as well as between *véraison* beginning and *véraison* end (for both correlations *r* = 0.8 and 0.7, in the core collection and the progeny, respectively). We observed strong positive correlations for budburst and flowering beginning in both populations (*r* = 0.7 and 0.6 in the core collection and the progeny, respectively) and for the pairs budburst–*véraison* beginning and *véraison* beginning–ripening in the core collection (*r* = 0.6 and 0.7, respectively). Furthermore, the timing of *véraison* end and ripening was highly correlated in the core collection (*r* = 0.75). Associations of total fertility index with timing of phenological events were slightly negative and averaged around *r* = −0.2; however, these were significant only in the core collection (Table 2). We did not consider correlations observed in only 1 year, as well as discordant correlations over different years.

**Table 2:** Average significant Spearman correlation between the phenological subtraits and fertility in the grapevine “phenological core collection” (below diagonal, *p* < 0.01) and the SY × PN progeny (above diagonal, *p* < 0.05)

Trait	BB	FB	FE	VB	VE	R	TF
BB		0.564	0.456	0.246	0.198	*NS*	−0.166^a^
FB	0.660		0.682	0.305	0.293	*NS*	*NS*
FE	0.663	0.823		0.292	0.331	*NS*	*NS*
VB	0.580	0.601	0.574		0.660	0.448	*NS*
VE	0.505	0.537	0.504	0.820		0.420	*NS*
R	0.356	0.494	0.465	0.682	0.751		*NS*
TF	*NS*	−0.256	−0.228	−0.286	−0.228	−0.230	

*TF* total fertility index, *BB* budburst, *FB* flowering beginning, *FE* flowering end, *VB* *véraison* beginning, *VE* *véraison* end, *R* ripening

^a^Significant in 2 years only

### QTL detection

Integrating the phenotype and genotype data from the Syrah and Pinot Noir progeny allowed us to detect nine QTLs related to phenological and fertility traits within six grapevine chromosomes: one for budburst (BB) on chromosome 15, one for flowering beginning (FB) on chromosome 7, five for *véraison* (VB, VE) on chromosomes 2, 15 and 17, and two for fertility (TF) on chromosomes 3 and 18 (Table 3). In the case of QTLs for phenology, both types of phenotype datasets, i.e. the one based on DOY and the other based on GDD were compared and gave similar results. We discovered three strong QTLs for *véraison* beginning with LOD scores exceeding genome-wide significance levels in two to five growing seasons. The locus on chromosome 2 was detected using datasets from five consecutive years and explained 11.3–21.0 % of phenotypic variation. Two interesting QTLs, located on chromosomes 15 and 17, explained 13.5–18.3 % and 7.9–14.2 % of trait variation, respectively. For *véraison* end two regions were discovered on chromosomes 2 and 15, which overlapped with the QTLs detected for *véraison* beginning. These two loci explained 14.0–43.7 % and 10.6–18.2 % of phenotypic variation, respectively. The other suggestive QTLs were identified for flowering beginning on chromosome 7, explaining 8.3–11.0 % of trait variation, and for budburst on chromosome 15, explaining 7.9–10.7 % of phenotypic variation and overlapping with the region discovered for *véraison* beginning. In the case of fruitfulness, the phenotypic data were surveyed over six consecutive years (2006–2011). This allowed us to identify two suggestive regions on chromosomes 3 and 18, consistent in two to three seasons and explaining 17.0–20.1 % and 8.3–9.1 % of phenotypic variation, respectively. We did not detect any stable QTL using datasets of flowering end and ripening.

**Table 3:** Main QTLs for phenology and fertility subtraits identified using phenotypic data collected during at least 2 years

Trait	Chr.	Associated marker	cM	Dataset	LOD Max	5 % Chr.	5 % GW	KW test (*p*)	% variance explained	Marker 1	Marker 2	CI (cM)	CI (bp)
BB	15	SNP15017	25.99	2008	2.93	2.7	4.3	*NS*	7.9	SNP7251	SNP6095	23.22–26.43	10,306,970–11,049,526
				2009	3.69	2.7	4.8	0.1	10.7				
FB	7	SNP5022	42.94	2008	4.15	2.9	4.4	0.0005	11.0	VMC8D11	SNP4094	40.91–46.62	16,401,285–17,046,939
				2009	3.24	2.9	4.5	0.005	8.3				
		VMC8D11	40.91	2010	3.29	2.9	4.4	0.05	9.2	SNP6082	SNP5022	39.98–42.94	16,871,868–17,131,388
VB	2	SNP4067	15.66	2007	9.08	2.8	4.5	0.0001	21.0	UDV27	VMC6F1	8.66–18.76	3,117,421–4,925,368
			17.01	2009	10.32	2.5	4.3	0.0001	20.5				
		VMC6F1	18.76	2006	4.69	2.8	4.5	0.005	12.9	SNP4067	2010D07R	17.01–27.20	4,703,600–5,737,052
			21.76	2010	5.87	2.9	4.3	0.0001	13.2				
		SNP4123	31.31	2008	5.82	2.7	4.9	0.001	11.3	2010D07R	VMC2C10.1	27.20–33.62	5,737,052–8,284,867
	15	SNP6063	18.10	2007	6.92	2.8	4.5	0.0001	17.1	SNP6116	SNP7251	15.10–23.22	10,306,970–12,346,018
		SNP7251	21.51	2010	4.92	2.7	4.3	0.0001	13.5	SNP6063	SNP4061	18.51–27.82	10,126,670–12,036,121
		SNP4061	27.82	2008	8.12	2.6	4.9	0.0001	18.3	SNP7251	VVIP33	23.22–33.31	9,926,097–10,306,970
				2009	5.63	2.6	4.3	0.0001	13.8				
	17	SNP8084	39.47	2009	3.29	2.8	4.3	*NS*	7.9	SNP8071	SNP7177	37.48–45.43	5,006,342–6,265,765
				2010	6.20	2.8	4.3	0.1	14.0				
		SNP8021	51.92	2007	3.68	2.9	4.5	0.0005	9.8	SNP7177	SNP7270	45.43–57.47	3,954,375–5,047,079
			52.92	2008	5.63	2.7	4.9	0.0005	14.2				
VE	2	1074J14R	27.61	2010	5.68	2.6	4.3	0.05	14.0	2010D07R	VMC2C10.1	27.20–33.62	5,662,834–8,284,867
		SNP4045	45.33	2007	16.33	2.8	4.5	0.0005	30.8	SNP8148	VMC7G3	41.00–49.48	9,207,951–18,083,459
		SNP7234	45.95	2009	9.13	2.8	4.4	0.0001	22.8				
		SNP7054	46.58	2008	14.90	2.7	4.4	0.0005	43.7				
	15	SNP6116	15.10	2010	5.21	2.7	4.3	0.0001	14.0	SNP0056	SNP6063	12.50–18.51	12,020,146–12,644,676
			17.10	2007	6.74	2.8	4.5	0.0001	18.2				
		SNP6063	18.10	2008	3.58	2.6	4.4	0.01	10.6	SNP6116	SNP7251	15.10–23.22	10,306,970–12,346,018
TF	3	SNP5050	21.12	2006	5.41	2.8	4.9	0.005	20.1	SNP3031	SNP7132	16.57–22.67	7,184,057–7,777,664
			20.12	2008	3.89	2.7	4.4	0.05	17.0				
	18	SNP7048	59.39	2009	3.37	3.0	4.3	0.05	9.1	VVIN83	SNP3032	51.39–63.23	9,543,849–12,480,055
			59.54	2010	3.20	3.0	4.4	0.1	8.5				
		SNP7055	60.54	2011	3.04	2.9	4.4	0.05	8.3	SNP7048		59.54–63.23	10,864,719–12,480,055

Position of the SSR and SNP markers flanking the QTL confidence intervals (CI) is based on the reference genome Pinot Noir clone ENTAV115 (Velasco et al. 2007)

### Candidate genes for development and fertility

We further focused on the QTLs related to budburst and *véraison* on chromosome 15, as well as to fertility on chromosomes 3 and 18. The number of functionally annotated genes in the QTL confidence intervals ranged from 89 (chromosome 3) to 210 (chromosome 18; supplementary Tables S3, S4 and S5). Among these, we selected and discussed several candidate genes that may contribute to grapevine development and fruitfulness, including genes essential for cell growth and coding for transcription factors and signalling molecules (Table 4).

**Table 4:** Candidate genes for grapevine phenology and fertility annotated in the PN40024 genome sequence

Chr.	Functional category	Gene annotation	Gene unique ID	Position	References
15	Metabolism	Chalcone and stilbene synthase VvCHS2	VIT_15s0021g02170	13,099,190–13,100,695	Parage et al. (2012)
		Glutathione S-transferase Z2 (GSTZ2)	VIT_15s0048g00950	15,085,283–15,090,953	Edwards et al. (2000); Braidot et al. (2008)
	Regulation	Scarecrow transcription factor 6 (SCL6)	VIT_15s0048g00270	14,396,828–14,399,794	Llave et al. (2002); Unver et al. (2010)
		Homeodomain GLABROUS1 (HDG1)	VIT_15s0048g02000	16,133,315–16,138,857	Nakamura et al. (2006)
	Signalling	Beta expansin VvEXPB3	VIT_15s0021g02670	13,673,378–13,674,771	Cosgrove (2000); Dal Santo et al. (2013b)
		Beta expansin VvEXPB4	VIT_15s0021g02700	13,735,914–13,737,731	
3	Cellular process	Mitotic checkpoint protein BUB3	VIT_03s0091g00710	7,149,908–7,160,638	Caillaud et al. (2009)
		Xyloglucan:xyloglucosyl transferase (XTH10)	VIT_03s0088g00650	8,842,942–8,844,322	Nunan et al. (2001); Bourquin et al. (2002);
	Metabolism	Sinapoylglucose:malate sinapoyltransferase (SMT)	VIT_03s0091g01200	7,855,565–7,861,870	Lehfeldt et al. (2000); Bienert et al. (2012)
		Serine carboxypeptidase S10	VIT_03s0091g01240	7,903,958–7,907,570	
		Sinapoylglucose-choline O-sinapoyltransferase (SCT)	VIT_03s0091g01270	7,935,398–7,938,767	
		Serine carboxypeptidase S10	VIT_03s0091g01290	7,951,175–7,955,690	
		Serine carboxypeptidase SCPL17	VIT_03s0088g00160	8,195,668–8,197,771	
		Serine carboxypeptidase S10	VIT_03s0088g00260	8,252,549–8,257,129	
	Signalling	Phytosulfokine PSK2	VIT_03s0088g00290	8,315,170–8,315,924	Motose et al. (2009)
18	Cellular process	Endo-1,4-beta-glucanase	VIT_18s0001g14040	12,086,327–12,090,970	Nunan et al. (2001); Buchanan et al. (2012)
	Regulation	Zinc finger (CCCH-type) family protein	VIT_18s0001g15570	13,682,439–13,700,040	Schmitz et al. (2005); Wang et al. (2008)
	Signalling	Cytokinin dehydrogenase 5 precursor	VIT_18s0001g13200	11,256,653–11,261,569	Fortes et al. (2011)
		Calmodulin binding protein, IQD32	VIT_18s0001g13870	11,862,607–11,871,541	Kline et al. (2010)
		Clavata 1 receptor kinase (CLV1)	VIT_18s0001g14610	12,668,387–12,671,744	Clark et al. (1997); Durbak and Tax (2011)
		ABA-responsive element-binding protein 3 (AREB3)	VIT_18s0001g14890	12,936,974–12,937,903	Kline et al. (2010)

## Discussion

### Phenotypic evaluation

Phenology and fertility were evaluated in two populations of cultivated grapevine with a potentially different level of variation: the germplasm collection and the offspring derived from a cross between two cultivars Syrah and Pinot Noir (the QTL mapping population). While we observed that the distributions of total fertility, budburst and timing of ripening had a similar shape in both populations, the distribution of flowering and *véraison* time in the mapping population generally displayed narrow peaks with many individuals finishing the particular developmental stage close to the average timing (Fig. 1b–e). Differences in the range of timing between the core collection and the QTL mapping population were more pronounced with the dates of *véraison* and ripening than with budburst or flowering (i.e. the DOY ranges of *véraison* beginning, *véraison* end and ripening were around twice larger for the core collection than for the mapping population, while the dates of budburst and flowering were similar in both populations). This could be explained by high phenotypic diversity in the core collection, resulting from the presence of specific accessions, such as the early ripening Nektar and the late ripening Ohanés (Supplementary Table S1).

The pairwise Spearman correlations between traits in this study confirmed that as the plants continue the growth cycle, each next event is more strongly correlated to the previous event. In former studies, it was observed that correlations between *véraison* and ripening dates were very high (Jones et al. 2005; Bock et al. 2011; Tomasi et al. 2011). However, in those studies budbreak was not significantly correlated with successive stages of development and thus it was regarded as an event influenced by the variable weather conditions early in the season. We, in turn, observed that the timing of budburst in both analysed populations was significantly correlated with the timing of flowering and the start of *véraison* (Table 2). This may suggest that genes underlying these traits are located within the same QTL regions.

The total fertility index in the present research was obtained by dividing the total number of fruit clusters by the total number of shoots per plant. It seems that climatic conditions during the observed growing seasons had an effect on the values of this parameter for all individuals in the core collection and in the segregating progeny of Syrah × Pinot Noir. Former comparative studies of fertility among grapevine varieties indicated that the differences in fruitfulness could be due to variation in cultivars, environmental factors (especially air temperature), as well as grafting and training methods (Sommer et al. 2000). Furthermore, this trait may be subjected to the growing conditions of the plant during the previous season. For instance, compared to well-exposed shoots, shoots which develop in dense shade are more likely to have nodes with less fruitful shoots during the following season (Sánchez and Dokoozlian 2005). Fertility, however, may also be affected by other factors, such as the number of flower clusters on the plant and the number of buds which were left after dormant pruning (Sansavini and Fanigliulo 1998; Morris and Main 2010). Based on our observations in the present survey during three and six growing seasons (in the core collection and the progeny population, respectively), it can be concluded that the studied trait is not genetically stable and depends on external conditions. All the plants in our experimental fields were pruned uniformly in each season; nevertheless, it may be noticed that there were quite high differences in rainfall and temperature means as well as flowering time during the analysed years, which might have affected fruitfulness (Supplementary Tables S1 and S2). The significant differences among seasons were revealed for both populations by the ANOVA test (data not shown).

### QTL detection and selection of candidate genes

The QTL mapping methods typically rely on the assumption that the phenotype follows a normal distribution for each QTL genotype. In our case, almost all phenotype datasets displayed a non-normal distribution. In general, the interval mapping procedure (including the multiple QTL model and cofactor selection) is quite robust against deviations from normality (van Ooijen 2009). Therefore, we performed this method together with a maximum likelihood mixture model and the permutation test based on the actual data, rather than assuming normality. We further tested if the results of interval mapping were not influenced by non-normal distributions of the data using the non-parametric Kruskal–Wallis analysis. With these approaches, we were able to detect nine QTLs on chromosomes 2, 3, 7, 15, 17 and 18.

The QTL for flowering time on chromosome 7 has already been found using the progeny from a cross between Gewürztraminer and Riesling (Duchêne et al. 2012). This locus contains several genes implicated in the flowering process, such as *VvFT* (*FLOWERING LOCUS T*) and *VvSVP1* (*SHORT VEGETATIVE PHASE 1*) (Carmona et al. 2007; Diaz-Riquelme et al. 2009). Other QTLs for this trait were found in progenies from different biparental crosses on chromosomes 1, 2, 6, 14, 15 and 18 (Costantini et al. 2008; Carreño Ruiz 2012; Duchêne et al. 2012). Likewise, the QTLs for *véraison* beginning and *véraison* end on chromosome 2 have been discovered previously in different studies, along with other QTLs on chromosomes 1, 3, 5, 6, 16 and 18 (Costantini et al. 2008; Carreño Ruiz 2012; Duchêne et al. 2012). The region discovered on chromosome 2 co-localized with the locus responsible for berry colour, which carries genes *VvMybA1* and *VvMybA2* involved in the regulation of anthocyanin biosynthesis (Kobayashi et al. 2004; Fournier-Level et al. 2010). The QTL on chromosome 17 has also been detected in the progeny of Ruby Seedless × Moscatuel as related to *véraison* and berry colour (Carreño Ruiz 2012). Here, we focused on the QTLs which have not been investigated yet. Below, we discuss several candidate genes underlying the QTLs for budburst and *véraison* located on chromosome 15, and for fertility on chromosomes 3 and 18 (Table 4).

On chromosome 15 we identified two overlapping QTLs related to *véraison* beginning and *véraison* end. These two intervals overlapped as well with the QTL for the start of budburst. Some other QTLs for budburst have recently been detected on chromosomes 4, 12 and 19 in different biparental populations (Carreño Ruiz 2012; Duchêne et al. 2012).

Among the genes on chromosome 15, we found several transcription factors implicated in bud and fruit development. For example, a plant-specific scarecrow-like transcription factor 6 (*SCL6*) is a member of the GRAS gene family, which controls a wide range of developmental processes, including hormone signalling and bud formation (Llave et al. 2002; Unver et al. 2010; Schulze et al. 2010). Another gene *HDG1* belongs to the class IV HD-ZIP gene family. Some genes of this family are involved in epidermal development and accumulation of anthocyanin in the shoot (Nakamura et al. 2006).

Cell elongation is an important process in plant development and it is driven by cell wall loosening and turgor pressure. Cell wall remodelling depends on a complex association of physical, chemical and enzymatic processes that are controlled by hormones and environmental factors. One group of genes regulating cell wall architecture is the expansin family. Two expansin genes are located in this QTL region: *VvEXPB3* and *VvEXPB4* (Cosgrove 2000; Dal Santo et al. 2013b).

Other candidate genes for *véraison* time in this interval include *VvCHS2*, which catalyzes the first step of flavonoid biosynthesis (Parage et al. 2012), and *GSTZ2* coding for glutathione S-transferase, which may be involved in the transport of flavonoids (Edwards et al. 2000; Braidot et al. 2008).

Several QTLs for fertility (fruitfulness) in grapevine were previously detected on chromosomes 5, 8 and 14 (Fanizza et al. 2005; Doligez et al. 2010). Here, we identified two QTLs for fertility, which were stable in at least two growing seasons: on chromosomes 3 and 18. Among the genes on chromosome 3, we found several with highly enriched GO terms, such as a cluster of genes coding for serine carboxypeptidase-like peptides (SCP, SCPL) (Table 4). These genes have been isolated from several plant species and some of them function as acyltransferases and lyases. They may be involved in a broad range of biochemical pathways, including those of secondary metabolite biosynthesis, herbicide conjugation and germination-associated degradation of seed protein reserves. Thus, they may be vital for normal plant growth and development, synthesis of compounds that protect plants against UV light and pathogens and resistance to natural and artificial xenobiotics (Lehfeldt et al. 2000; Fraser et al. 2005; Bienert et al. 2012). In grapevine, genes from this cluster on chromosome 3 have been identified as candidate glucose-acyltransferases (*VvGAT*-*like*) acting in the proanthocyanidin biosynthetic pathway. Proanthocyanidins are secondary metabolites belonging to flavonoids, which play a major role in plant protection against biotic and abiotic stresses (Carrier et al. 2013). As such, variation in these genes might influence grapevine fertility.

This QTL region harbours another key gene required in many aspects of cell wall biosynthesis: *XTH*, a member of xyloglucan endotransglycosylases/hydrolases. These enzymes are active in xylem and phloem fibres at the stage of secondary wall formation. They reconstruct primary walls, probably by creating and reinforcing the connections between the primary and secondary wall layers, and these cross-links may play an important role in preventing further cell expansion (Bourquin et al. 2002).

Other candidate molecules in this region include the cell cycle arrest protein BUB3, which functions in a molecular complex controlling cell division (Caillaud et al. 2009) and a precursor of phytosulfokine (PSK2), which is a sulfated peptide hormone required for the proliferation and differentiation of plant cells (Motose et al. 2009).

When searching amongst the 210 genes on chromosome 18, we found several candidate molecules potentially contributing to grapevine fruitfulness, such as transcriptional factors. For example, one candidate gene in this region belongs to a CCCH-type zinc finger family and members of this family have been shown to play diverse roles in plant developmental processes and environmental responses, e.g. in the physiological control of female fertility at the level of early embryonic development (Schmitz et al. 2005; Wang et al. 2008).

There are several genes in this interval which are involved in the response to abscisic acid (ABA). ABA is a hormone that controls the overall plant response to environmental stresses. This molecule influences also the onset of grape berry ripening (Gambetta et al. 2010). Transcription factor AREB3 is an ABA response element DNA-binding protein, which is required in initiating the long-term changes in gene expression induced by ABA (Kline et al. 2010). Calmodulin binding protein IQD32 is a phosphopeptide significantly altered in response to ABA treatment. Calcium signalling plays an important role in plants for coordinating a wide range of developmental processes and responses to environmental change. The frequently predicted nuclear localization of IQD proteins suggests that they link calcium signalling pathways to the regulation of gene expression (Abel et al. 2005; Kline et al. 2010). For example *SUN*, a member of the IQD gene family in tomato, influences floral and fruit morphology (Wu et al. 2011).

Also cytokinins are essential plant hormones that control various aspects of plant growth and development, such as cell division and flower and fruit formation. These molecules are involved in berry set and in growth promotion, and tend to inhibit ripening. One of the candidates located on chromosome 18 codes for a cytokinin dehydrogenase, active in the maintenance of optimal cytokinin concentration via their degradation (Fortes et al. 2011).

Another key regulator of cellular events, which is located in this QTL, is the LRR receptor kinase *CLAVATA1* (*CLV1*). This gene and its homologues are involved in plant development and environmental responses. CLV1 may function as a signal transduction component that acts in the communication of cell division. In *Arabidopsis*, this kinase plays a critical role in the maintenance of the stem cells in shoot apical meristems and regulates fruit development (Clark et al. 1997; Durbak and Tax 2011).

The last proposed candidate in this interval, Endo-(1,4)-β-glucanase, has been implicated in the breakdown of cell walls during processes observed in normal growth and development, including floral abscission and fruit ripening (Nunan et al. 2001; Buchanan et al. 2012).

## Conclusions

In the present research, we compared phenotypic variation in phenology and fertility in two grapevine populations: (1) the collection of different major and minor grapevine cultivars, selected over years and grown locally or worldwide for fruit production and (2) the progeny derived from a new experimental cross between two varieties, Syrah and Pinot Noir, maintained in the field for several years. Although these two populations have different degrees of relationship among individuals, we found that the range of phenotypic variation for our traits of interest in the progeny population covered in the most part the variation recorded for the core collection, which was selected specifically to maximize the variability in the timing of key developmental stages. In addition, as expected, we detected several accessions in the core collection with the earlier or later dates for budburst, flowering and harvest, compared to the progeny population. Nevertheless, the SY × PN progeny could be used as a QTL mapping population to identify loci explaining this phenotypic variation. For the phenological traits, we tried to isolate the genetic and climatic influence (namely, air temperature) by using the DOY, as well as the GDD phenotypic dataset. Ultimately, we identified nine minor and major QTLs related to budburst, flowering beginning, *véraison* beginning, *véraison* end and total fertility. The genomic region on chromosome 15 contains overlapping QTLs for budburst and *véraison*, and harbours genes underlying fruit development, including expansins (*VvEXPB3, VvEXPB4*) and enzymes involved in biosynthesis and transport of flavonoids (*VvCHS2*, *GSTZ2*). For fertility, two QTLs were located on chromosomes 3 and 18. Among the genes potentially affecting grape fruitfulness are the complex of serine carboxypeptidase-like genes (*SCPL*), xyloglucan:xyloglucosyl transferase *XTH10*, protein kinase *CLV1* and ABA-responsive molecules (*AREB3*, *IQD32*). The next step will be to study polymorphism within these candidate loci and to further investigate their relationship with trait variation by analysing multiple accessions from the core collection. The long-term objective of this research is to provide information on the genetic basis of these traits and to facilitate selection of varieties adapted to atmospheric conditions of a specific geographic region.

## Electronic supplementary material

Below is the link to the electronic supplementary material.

Supplementary material 1 (XLSX 222 kb)
